# Covariation of gene frequencies in a stepping-stone lattice of populations^1^

**DOI:** 10.1016/j.tpb.2014.12.004

**Published:** 2014-12-23

**Authors:** Joseph Felsenstein

**Affiliations:** Department of Genome Sciences and Department of Biology, University of Washington, Box 355065, Seattle, WA 98195-5065

**Keywords:** Stepping-stone model, genetic drift, neutral mutation, migration, geographic differentiation

## Abstract

For a one- or two-dimensional lattice of finite length consisting of populations, each of which has the same population size, the classical stepping-stone model has been used to approximate the patterns of variation at neutral loci in geographic regions. In the pioneering papers by Maruyama (1970a, 1970b, 1971) the changes of gene frequency at a locus subject to neutral mutation between two alleles, migration, and random genetic drift were modeled by a vector autoregression model. Maruyama was able to use the spectrum of the migration matrix, but to do this he had to introduce approximations in which there was either extra mutation in the terminal populations, or extra migration from the subterminal population into the terminal population. In this paper a similar vector autoregression model is used, but it proves possible to obtain the eigenvalues and eigenvectors of the migration matrix without those approximations. Approximate formulas for the variances and covariances of gene frequencies in different populations are obtained, and checked by numerical iteration of the exact covariances of the vector autoregression model.

Stepping stone models of migration on rectangular lattices of populations have become of increasing interest as samples of many SNP loci have been collected in contiguous geographic areas, particularly in human populations. Stepping-stone models were pioneered independently by Malécot (1951) and by Kimura (1953; Kimura and Weiss, 1964). They considered lattices of infinite numbers of populations connected by migration in one and two dimensions, and derived expressions for the genetic variability expected in the populations in a balance between mutation and genetic drift of neutral alleles.

Lattices of finite size are of greater practical interest as models of real populations. Malécot (1948, 1950) pioneered them, using a model of a torus or circle of a finite number of populations. Maruyama (1970a, 1970b, 1971) was the first to consider stepping stone models for ordinary one- and two-dimensional linear or rectangular lattices of finite numbers of populations. He gave expressions for the variances and covariances of gene frequencies for arbitrary pairs of populations when there was a two-allele neutral mutation model in which the population had reached its equilibrium distribution. To do so he used an approximate model which had some lack of realism in the treatment of migration into the terminal populations of the lattice. He considered different variations in the way these terminal populations were modeled.

In the present paper I will treat a more exact model with a more realistic pattern of migration into the terminal populations. The results are similar, but not identical, to Maruyama's results.

Patterson et al. (2006) have pointed out the importance of using the eigenvectors and eigenvalues of the variation in gene frequencies in principal components analysis of data from different populations, an approach which goes back to Menozzi et al. (1978) and is reviewed by Cavalli-Sforza and Feldman (2003). An important issue is the interpretation of any significant patterns of geographic differentiation that are found. They do not necessarily indicate historical events such as waves of migration.

Novembré and Stephens (2008) have pointed out that the eigenvectors of a lattice model of migration are startlingly similar to principal components found for gene frequency patterns in geographic studies of genetic variation. When one of these principal components is seen, this makes it less obvious that it must arise from an historical invasion event, as the stepping stone models do not include historical invasions. They pointed out that migration matrices such as the ones Maruyama used fall into the class of Toeplitz matrices (Gray, 2006) for which the eigenvectors and eigenvalues can readily be computed. The more exact model which we use here has migration matrices that are not precisely Toeplitz matrices. It turns out that their eigenvalues have similarities with those of Maruyama's matrices, and their eigenvectors are readily found.

The immediate purpose of obtaining expressions for the covariances will be to approximate the joint distribution of gene frequencies in the populations by a multivariate normal distribution, for which the expectations and covariances are all that we need to determine the distribution. The expectations are easy to obtain; the covariances do still require one approximation but, when checked against an exact numerical solution of the equations, seem closer to the correct values than Maruyama's approximations are. The joint distribution of gene frequencies will not actually be normal, but for cases with small departures of gene frequencies from their expectations it will come close to being multivariate normal. Elsewhere I hope to discuss the development of an approximate maximum likelihood inference of the parameters of the model using this approximation. The formulas developed here may also be useful to others working with finite stepping-stone models who wish to have a closer approximation of the covariances of gene frequencies than has hitherto been available.

## 1. The model

The finite stepping-stone model is a linear lattice of *n*_1_ populations (if in one dimension) or a rectangular lattics of *n*_1_ × *n*_2_ populations (if in two dimensions). Analogous models can be erected in higher numbers of dimensions. Each population has the same size, *N* individuals. The model has discrete, nonoverlapping generations. In each generation an infinite number of offspring are produced in each population. From these a random *N* are chosen to survive to be the adults of the next generation. The population sizes thus remain constant.

Each individual among the offspring is diploid, and has two parents. Migration enters the picture in the locations of the parents. For most of the populations in a one-dimensional lattice, there is a probability 1 − *m* that a particular individual drawn to be a parent comes from the same population. In a one-dimensional lattice there is a probability $\frac{1}{2}m$ that this parent comes from the population to the left, and a probability $\frac{1}{2}m$ that it comes from the population to the right. The exception is when the offspring population is the first or the last in the lattice. Then there is a probability $\frac{1}{2}m$ that it comes from the adjacent population, and the rest of the time, $1-\frac{1}{2}m$ of the time, it comes from the same population.

**Figure 1:**
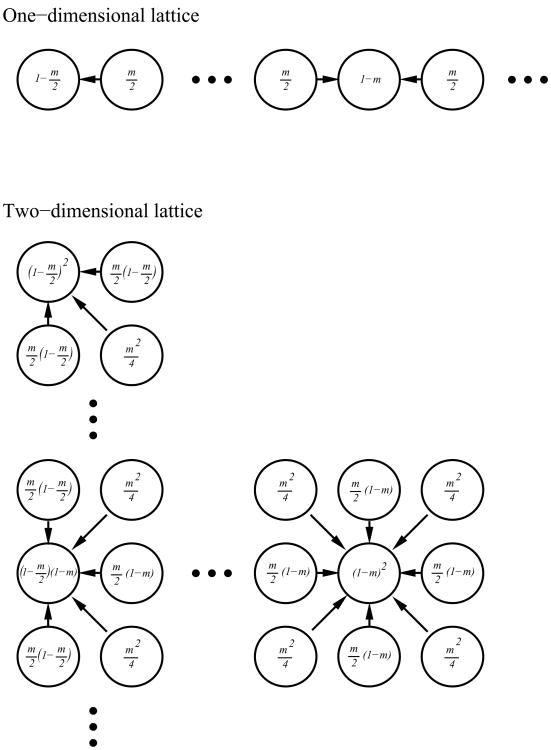
Diagram of the migration pattern in a one- and two-dimensional stepping stone model. In each case the recipient population is shown together will all populations contributing migrants to it, with the fraction of the recipient population coming from each of these populations shown. This is shown for an interior population and a terminal population for the one-dimensial stepping stone model, and for an interior population, a side population, and a corner population for a two-dimensional stepping stone model.

If the lattice is a two-dimensional one, the same process is imagined to occur in both dimensions, completely independently. Thus for a population that is not on a boundary of the lattice, a parent for the (*i*, *j*) population may have come from that population, or from any of the 8 populations surrounding that one, as shown in Figure 1. It can have come from the four populations adjacent on diagonals with probability $\frac{1}{4}m^{2}$ each, and from the four populations adjacent in one direction with probability $\frac{1}{2}m(1-m)$ each. With probability (1 − *m*)^2^ it comes from population (*i*, *j*).

If the population is on one of the boundaries of the two-dimensional lattice, but not at a corner, its parents come from the two adjacent side populations with probability $\frac{1}{2}m(1-\frac{1}{2}m)$ each, from the adjacent interior population with probability $\frac{1}{2}m(1-m)$, from the two nearest diagonal populations with probability $\frac{1}{4}m^{2}$ each, and hence from the same population the remaining $\left(1-\frac{1}{2}m\right)$ (1 − *m*) of the time. When the population is in a corner of the lattice, a parent comes from the two nearby populations with probability $\frac{1}{2}m(1-\frac{1}{2}m)$ each, from the one population one step away on the diagonal with probability $\frac{1}{4}m^{2}$, and from that population itself with the remaining probability ${\left(1-\frac{1}{2}m\right)}^{2}$.

These seemingly complicated patterns are really just the consequence of having $\frac{1}{2}m$ come from each neighboring population in one dimension, with the two-dimensional pattern being independent movement in the two dimensions.

Figure 1 shows the migration rates in the present model in one- and two-dimensional lattices. This is slightly different from the migration pattern usually used in two-dimensional stepping-stone models. In most previous work, in two dimensions the migration can only come from one of the two populations immediately adjacent in one dimension, or immediately adjacent in the other dimension. Migrants could only come from populations (*i* − 1, *j*), (*i* + 1, *j*), (1, *j* − 1), or (*i*, *j* + 1). The present scheme, in which migration occurs or not in either dimension, independently, leads to greater mathematical tractability. It is worth noting that such a scheme is also implicit in Maruyama's papers.

The model follows the gene frequency of one allele at a locus, in the presence of migration, mutation, and genetic drift. The model of mutation has two alleles with mutation back and forth between them. If the mutation rate from *A* to *a* is *μ*, and the mutation rate from *a* to *A* is *ν*, the equilibrium gene frequency is *p̄* = *ν*/(*μ* + *ν*). Mutation in such a one-locus model acts as if it were a form of migration. If we set an additional rate of migration into each population of *m*_∞_ = *μ* + *ν*, and have these immigrant copies of the gene be drawn from a pool in which the gene frequency of *A* is *p̄*, this will be indistinguishable from a model that has migration plus mutation between two alleles.

In the present model, parents are chosen according to the migration model, with the two parents of an individual independently drawn. Each parent contributes an allele to the offspring. Mutation occurs (or does not) for each copy of the gene. Each population thus has a pool of newborn offspring, whose genetic composition is characterized by the gene frequency of the *A* allele (which I will call *p*). Among these offspring, the model makes the presence or absence of the *A* allele at each copy from that pool independent of the other copies, so that we do not need to concern ourselves with the diploid genotype frequencies in the pool.

Genetic drift occurs by sampling *N* diploid individuals from the offspring pool, without replacement. As the presence of the *A* allele in each copy in the diploid individuals is independent, this has the same effect as drawing 2*N* times from a pool which has gene frequency *p*.

We can take the gene frequencies in the local populations and arrange them in a column vector, whose length is the number of populations. For the two-dimensional case this involves taking the gene frequencies in the rows of the array of populations, forming each into a column vector, and stacking them on top of each other, so that the first two entries in the vector are the gene frequencies for populations (1, 1) and (1, 2). Let the populations now be numbered in the order in which they appear in this vector.

For both one- and two-dimensional cases we will see below that we can use the migration pattern to create a migration matrix **M** whose elements *m_ij_* are the probability that a copy of the gene found in the newborn offspring pool for population *i* came from a parent which was in population *j*, we can then write for population *i* the gene frequency in the next generation:

(1) $$p_{i}^{\prime }=(1-m_{\infty })\sum_{j=1}^{n}m_{ij}p_{j}+m_{\infty }\bar{p}+\varepsilon_{i}$$

where *ε_i_* is the change due to genetic drift. The random variable $p_{i}^{\prime }$ is a binomial proportion after 2*N* trials with a probability of success equal to the sum of the first two terms on the right-hand side of this equation. The expectation of *ε_i_* is thus zero.

This set of equations can be put into matrix form as

(2) $$p^{\left(t+1\right)}=(1-m_{\infty })Mp^{\left(t\right)}+m_{\infty }\bar{p}1+\varepsilon^{\left(t\right)},$$

where the vector **1** is a column vector of 1's and **p**^(*t*)^ is the vector of gene frequencies in the populations in the adult stage of generation *t*. This equation is true for any pattern of recurrent migration, not just for stepping-stone lattices.

Note that, except for the error not being multivariate normally distributed, these equations are essentially a vector autoregression (VAR) model, beloved of econometricians. In this case they are (nearly) a VAR(1) model, as each generation depends only on the preceding one. In VAR models, the objective is to infer the regression coefficients from a series of observations. Here most of the regression structure is known, it is also known that the errors *ε_i_* do not covary, and the objective is to predict the variances and covariances of the model at equilibrium.

This model is close to the models used by Maruyama (1970a, 1970b, 1971) but is not exactly the same. For the terminal population he considered two different models. One (his “absorbing boundary”) had migration rate *m*/2 into the terminal population from the subterminal population, and also an extra inflow of *m*/2 into from a pool at the equilibrium frequency. This allowed a fraction 1 − *m* of the terminal population to be nonmigrants, which made all the diagonal elements of the migration matrix equal. The second (his “reflecting boundary”) (Maruyama 1970a, 1970b) had migration at rate *m* from the subterminal population into the terminal population, rather than *m*/2. Again the migration matrix had all its diagonal elements equal. We will not adopt either of these measures, hoping to be able to cope with a migration matrix that does not have all of its diagonal values equal. It is also worth noting that Maruyama's variable **p** is not our **p**, but is the deviation of the gene frequency from its equilibrium value.

## 2. Transformation to uncorrelated variables

In the cases we consider, the matrix **M** will be symmetric. For all cases of symmetric migration among populations (not just for stepping-stone lattices of populations) it will be possible to write it in terms of its eigenvalues and eigenvectors as

(3) $$M=U\Lambda U^{T}$$

where the matrix **U** of eigenvectors is orthonormal, so that its transpose **U***^T^* is also its inverse. The diagonal elements of the matrix of eigenvalues **Λ** will be real numbers. Substituting this form of **M** into equation (2) we get

(4) $$p^{\left(t+1\right)}=(1-m_{\infty })U\Lambda U^{T}p^{\left(t\right)}+m_{\infty }\bar{p}1+\varepsilon^{\left(t\right)},$$

where **1** is the column vector whose entries are all 1. This equation can be premultiplied by **U***^T^* to get

(5) $$U^{T}p^{\left(t+1\right)}=(1-m_{\infty })\Lambda U^{T}p^{\left(t\right)}+m_{\infty }U^{T}\bar{p}1+U^{T}\varepsilon^{\left(t\right)},$$

since **U***^T^* is the inverse of **U** so that their product is the identity matrix and thus disappears from the matrix products.

We now consider a new vector of random variables

(6) $$x^{\left(t\right)}=U^{T}p^{\left(t\right)}$$

and write the vector **U**^*T*^ *p̄***1** as **q**, which is a constant vector and not a random variable. We notice that since the expectations of the random variables $\varepsilon_{i}^{\left(t\right)}$ are zero, we can write the equation for **x**^(*t*)^ as

(7) $$x^{\left(t+1\right)}=(1-m_{\infty })\Lambda x^{\left(t\right)}+m_{\infty }q+\eta^{\left(t\right)}$$

The vectors of random variables ***η***^(*t*)^ will have expectation zero.

The first eigenvalue *λ*_1_ will be, for all the migration matrices we consider, 1, and its associated eigenvector will have all elements equal. The other eigenvectors will be orthogonal to this. It follows that **q** will have its first element nonzero, and all other elements zero.

## 3. Expectations

If we now take the expectations of the terms in equation (7), we can readily use equation (7), to show for each element of the vector **x** that in the long run that the process will approach a stationary distribution. In that distribution, the expectations the elements of $x_{i}^{\left(t\right)}$ are each equal to the expectations of the corresponding elements of **q**. This is asymptotically true for *t* very large, when the effect of the initial frequencies of the $p_{i}^{\left(0\right)}$ have been lost. Going back to the elements of the vectors **p**^(*t*)^ in this limiting distribution, these all have equal expectations, Going back to the elements of the vectors **p**^(*t*)^ in this limiting distribution, these all have equal expectations, which are simply *p̄*, the mutational equilibrium gene frequency. The equation for the expectations of the **x**^(*t*)^ in this limiting distribution, which will be called **x̄**^(*t*)^, is then

(8) $${\bar{x}}^{\left(t+1\right)}=(1-m_{\infty })\Lambda {\bar{x}}^{\left(t\right)}+m_{\infty }q.$$

We can subtract this equation termwise from equation (7) and obtain an equation for the deviation of the **x**^(*t*)^ from their expectations. If we call these deviations **y**^(*t*)^, this is then

(9) $$y^{\left(t+1\right)}=(1-m_{\infty })\Lambda y^{\left(t\right)}+\eta^{\left(t\right)}$$

It is easy to see that the expectations of the **y**^(*t*)^ are all zero. For this reason, the equation for the **y**^(*t*)^ is simpler than the equation for the **x**^(*t*)^, and we now use it to investigate the covariances.

## 4. Approximating the stochastic process

If we write, from equation (9), the expression for one of the elements of the vector **y**^(*t* + 1)^, it is

(10) $$y_{i}^{\left(t+1\right)}=(1-m_{\infty })\lambda_{i}y_{i}^{\left(t\right)}+\eta_{i}^{\left(t\right)}$$

If the elements of ***η***^(*t*)^ were all independent of each other, it would be easy to show that the $y_{i}^{\left(t\right)}$ would be independent random variables. Actually, the $\eta_{i}^{\left(t\right)}$ are not, strictly speaking, independent. The vector ***η***^(*t*)^ is a linear transformation of a set of independent binomial variates (each element of *ε*^(*t*)^ being the difference between a binomial frequency and its expectation). However the nonindependence is subtle and, for our purposes, unimportant. The objective of this paper is to derive the covariances of the gene frequencies, for use in a statistical inference when approximating the distribution of the gene frequencies as multivariate normal. It will be sufficient to be able to show that the *η_i_*^(*t*)^ are approximately uncorrelated.

To do this another approximation will also be made. The $\varepsilon_{i}^{\left(t\right)}$ have variances that arise in the binomial sampling of the gene frequencies in going from the infinite number of newborn individuals in a population to the finite number *N* of surviving adults. In any generation in which that gene frequency among the newborns is *p_i_*, the binomial variance on sampling 2*N* copies of the gene is *p_i_*(1 − *p_i_*)/(2*N*). At stationarity, the values of *p_i_* across different generations have expectation *p̄* and a variance, which we can call $\sigma_{i}^{2}$. The expectation of the binomial variance across generations can be written in terms of this as

(11) $$E[\frac{1}{2N}p_{i}(1-p_{i})]=\frac{1}{2N}(E[p_{i}]-E[p_{i}^{2}])=\frac{1}{2N}(\bar{p}-{\bar{p}}^{2}-\sigma_{i}^{2})$$

At this point we do not know the values of the quantities $\sigma_{i}^{2}$. They depend on the very thing we want to compute, the variances and covariances of the gene frequencies *p_i_*. There are likely to be differences between the variances $\sigma_{i}^{2}$, with terminal populations having a somewhat higher variance of gene frequency than interior populations.

In the limiting case when migration rates are large, the gene frequencies in all populations will be very similar. In that limit the variances $\sigma_{i}^{2}$ will be equal. The approximation made here will be to assume that we are near this limit, and that for the purposes of further calculation we can assume that all of the $\sigma_{i}^{2}$ are equal to the With that approximation, we can show that the random variables $\eta_{i}^{\left(t\right)}$ are not correlated. We have seen that they are the elements of the vector **U***^T^ε* ^(*t*)^. We know that the $\varepsilon_{i}^{\left(t\right)}$ have zero covariances, as they are independent random variables. If we use the approximation that the $\varepsilon_{i}^{\left(t\right)}$ have equal variances *σ*^2^, then we can write the covariance matrix of the $\eta_{i}^{\left(t\right)}$ as

(12) $$E[\eta \eta^{T}]=E[U^{T}\varepsilon \varepsilon^{T}U]=U^{T}E[\varepsilon \varepsilon^{T}]U=U^{T}(\frac{1}{2N}(\bar{p}(1-\bar{p})-\sigma^{2})I)U=\frac{1}{2N}(\bar{p}(1-\bar{p})-\sigma^{2})U^{T}U=\frac{1}{2N}(\bar{p}(1-\bar{p})-\sigma^{2})I$$

As this is diagonal, the covariances of different elements of ***η*** are thus, to close approximation, zero.

## 5. Covariances of gene frequencies

With the covariances of the $\eta_{i}^{\left(t\right)}$ now determined to (approximately) be zero, the random variables for the different *i* in equation (9) are now uncorrelated. They all have expectation zero. Its variance is easily determined by noting that the sum on the right-hand side is of two uncorrelated variables, whose covariance is therefore zero, so that:

(13) $$\text{Var}(y_{i}^{\left(t+1\right)})={\left(1-m_{\infty }\right)}^{2}\lambda_{i}^{2}\text{Var}(y_{i}^{\left(t\right)})+\text{Var}(\eta_{i}^{\left(t\right)})$$

Once this random process has reached stationarity, the variance of *y_i_* in all generations is equal and, with our approximation of $\sigma_{i}^{2}$ by *σ*^2^, we can then solve for the variance of *y_i_* as

(14) $$\text{Var}(y_{i})=\frac{\text{Var}(\eta_{i})}{1-{\left(1-m_{\infty }\right)}^{2}\lambda_{i}^{2}}=\frac{\bar{p}(1-\bar{p})-\sigma^{2}}{2N(1-{\left(1-m_{\infty }\right)}^{2}\lambda_{i}^{2})}.$$

The remaining covariances are all zero.

The covariances of the *x_i_* are the same as those of the *y_i_*, since these vectors differ by a constant vector. To obtain the covariances of the gene frequencies *p_i_*, we use the fact that the gene frequencies are a linear transformation of the *y_i_*. From equation (6),

(15) p=U(y+q),

so that since **q** is **U**^*T*^ *p̄***1**,

(16) $$p=Uy+\bar{p}1$$

so that

(17) $$p-\bar{p}1=Uy.$$

The covariances of **p** will then be

(18) $$E[(p-\bar{p}1){\left(p-\bar{p}1\right)}^{T}]=E[Uyy^{T}U^{T}]=UE[yy^{T}]U^{T}$$

From equation (14) we can now write the covariances of the *p_i_* as

(19) $$\text{Cov}[p]=U\;\text{diag}(\frac{\bar{p}(1-\bar{p})-\sigma^{2}}{2N(1-{\left(1-m_{\infty }\right)}^{2}\lambda_{i}^{2})})U^{T}$$

Since the diagonal matrix in this expression can also be written in terms of the square of the diagonal matrix of eigenvalues, it turns out that this covariance matrix can also be written in terms of the inverse of a simple function of the migration matrix:

(20) $$\text{Cov}[p]=(\bar{p}(1-\bar{p})-\sigma^{2}){\left(2N(I-{\left(1-m_{\infty }\right)}^{2}M^{2})\right)}^{-1}$$

Of course, the diagonal elements of the covariance matrices are all assumed to be equal to *σ*^2^. Although the above expressions compute *σ*^2^ in terms of itself, we will find a way to untangle that.

We now know that the expectations of the *p_i_* are all *p̄*, and for the (approximated) process we have the covariances in terms of the eigenvalues and eigenvectors of **M**. Now all we need is to find those. The derivation since (1) has been general for any system of recurring migration whose migration rates among populations are symmetric, provided that the migration is strong enough to allow us to assume that the variances $\sigma_{i}^{2}$ are nearly equal. Now we need to use the particular pattern of migration on a finite lattice to obtain the eigenvalues and eigenvectors of the migration matrix.

## 6. The spectrum of the migration matrix

The matrix in one dimension is

(21) $$M=[\begin{array}{ccccccc} 1-\frac{1}{2}m & \frac{1}{2}m & 0 & \cdots & 0 & 0 & 0\\ \frac{1}{2}m & 1-m & \frac{1}{2}m & \cdots & 0 & 0 & 0\\ 0 & \frac{1}{2}m & 1-m & \cdots & 0 & 0 & 0\\ \vdots & \vdots & \vdots & \ddots & \vdots & \vdots & \vdots \\ 0 & 0 & 0 & \cdots & 1-m & \frac{1}{2}m & 0\\ 0 & 0 & 0 & \cdots & \frac{1}{2}m & 1-m & \frac{1}{2}m\\ 0 & 0 & 0 & \cdots & 0 & \frac{1}{2}m & 1-\frac{1}{2}m \end{array}]$$

For the one-dimensional case we will use *n* in place of *n*_1_ for the number of populations, to reduce typographical stress.

Feller (1957, section XVI.3, pp. 389-390) treats a random walk with two reflecting boundaries, whose transition matrix is the above matrix for the case when *m* = 1. He uses a partial fractions method; as he notes, this is equivalent to a spectral decomposition of the matrix. His quantity *s_r_* is the reciprocal of the eigenvalue *λ*_*r* + 1_. Thus the eigenvalues for *m* = 1 are

(22) $$\lambda_{i}=\cos(\frac{(i-1)\pi }{n})$$

and if for *m* = 1 we call the matrix **R**, we have for general *m*

(23) M=(1−m)I+mR

so that the eigenvalues of **M** are the corresponding linear combinations which turn out to be

(24) $$\lambda_{i}=1-m(1-\cos(\frac{(i-1)\pi }{n}))$$

The eigenvectors of **M** will be the same as those of **R**. In the Appendix it is shown that if these are scaled so as to be orthonormal the right eigenvectors can be written as

(25) $${\left(U^{T}\right)}_{ij}=\sqrt{\frac{\Delta_{i}}{n}}\cos(\frac{(i-1)(2j-1)\pi }{2n}),\;i=1,2,\ldots ,n,\;j=1,2,\ldots ,n$$

where the quantity Δ*_i_* is 1 if *i* = 1 and 2 otherwise. Maruyama found a similar quantity necessary in the expressions for his eigenvectors.

By comparison, Maruyama's eigenvalues were

(26) $$\lambda_{i}=1-m(1-\cos(\frac{i\pi }{n+1})).$$

His eigenvectors could also be written in terms of cosines, but were different from the expressions for our matrix.

That there is a connection to Maruyama's matrix is made clearer if we consider a “shift matrix” **S** which has the diagonal above the main diagonal filled with 1s, and the rest of the elements zero:

(27) $$S=[\begin{array}{ccccccc} 0 & 1 & 0 & \cdots & 0 & 0 & 0\\ 0 & 0 & 1 & \cdots & 0 & 0 & 0\\ 0 & 0 & 0 & \cdots & 0 & 0 & 0\\ \vdots & \vdots & \vdots & \ddots & \vdots & \vdots & \vdots \\ 0 & 0 & 0 & \cdots & 0 & 1 & 0\\ 0 & 0 & 0 & \cdots & 0 & 0 & 1\\ 0 & 0 & 0 & \cdots & 0 & 0 & 0 \end{array}]$$

One can write Maruyama's matrix as $(1-m)I+\frac{1}{2}mS+\frac{1}{2}mS^{T}$. Our matrix **M** can also be written in terms of **S**, as

(28) $$M=I+\frac{1}{2}mS+\frac{1}{2}mS^{T}-\frac{1}{2}m(SS^{T}+S^{T}S).$$

## 7. Solving for the covariances

Using equation (19) together with equations (26) and (25) we can now write the covariance of populations in the one-dimensional case as

(29) $$\text{Cov}[p_{i},p_{j}]=\frac{1}{2N}(\bar{p}(1-\bar{p})-\sigma^{2})\sum_{k=1}^{n}\frac{\Delta_{k}}{n}\cos(\frac{(k-1)(2i-1)\pi }{2n})\cos(\frac{(k-1)(2j-1)\pi }{2n})\times \frac{1}{1-{\left(1-m_{\infty }\right)}^{2}{\left(1-m[1-\cos(\frac{(k-1)\pi }{n})]\right)}^{2}}$$

For the two-dimensional case we note that the migration matrix **M** is the result of independent migration in the two dimensions; it turns out that

(30) $$M=M^{\left(1\right)}⊗M^{\left(2\right)},$$

where the superscripts indicate the two dimensions and ⊗ is the Kronecker product of matrices. It is well-known for Kronecker products that if **Λ**^(1)^ and **Λ**^(2)^ are the diagonal matrices of the eigenvalues of (respectively) **M**^(1)^ and **M**^(2)^, then the diagonal matrix of eigenvalues of **M** is the Kronecker product **Λ**^(1)^ ⊗ **Λ**^(2)^, which is the diagonal matrix whose diagonal elements are all *n*_1_ × *n*_2_ products of one of the eigenvalues of **M**^(1)^ and one of the eigenvalues of **M**^(2)^. Similarly, the *n*_1_ *n*_2_ × *n*_1_ *n*_2_ matrix **U** of eigenvectors of **M** is the Kronecker product of the *n*_1_ × *n*_1_ matrix **U**^(1)^ of eigenvectors of **M**^(1)^ and the *n*_2_ × *n*_2_ matrix **U**^(2)^ of eigenvectors of **M**^(2)^.

If for the two-dimensional case we denote the gene frequency of the population at position (*i, j*) as *p_ij_*, the result is that

(31) $$\text{Cov}[p_{ij},p_{kl}]=\frac{1}{2N}(\bar{p}(1-\bar{p})-\sigma^{2})\sum_{g=1}^{n}\sum_{h=1}^{n}\frac{\Delta_{g}}{n}\frac{\Delta_{h}}{n}\cos(\frac{(g-1)(2i-1)\pi }{2n})\cos(\frac{(h-1)(2j-1)\pi }{2n})\times \cos(\frac{(g-1)(2k-1)\pi }{2n})\cos(\frac{(h-1)(2l-1)\pi }{2n})\times \frac{1}{1-{\left(1-m_{\infty }\right)}^{2}{\left(1-m[1-\cos(\frac{(g-1)\pi }{n})]\right)}^{2}{\left(1-m[1-\cos(\frac{(h-1)\pi }{n})]\right)}^{2}}$$

Higher numbers of dimensions can be accommodated in an exactly analogous way. The generalization of the two-dimensional case to having different migration rates *m*_1_ and *m*_2_ in the two dimensions is straightforward.

## 8. Solving for *σ^2^*

There is still the vexing matter of the variance *σ*^2^. One method of inferring it is to start with *σ*^2^ = 0 and use equations (29) or (31) to compute all the Cov[*i,i*]. Then from these new estimates of the $\sigma_{i}^{2}$, make a new estimate of *σ*^2^ by averaging these. One might want to continue this until the value of *σ*^2^ converges. It actually does not converge if 4*Nm* < 1. However if in each iteration we instead make a weighted average of two quantities, one the mean of the new $\sigma_{i}^{2}$ and the other the previous value of *σ*^2^, with their weights 4*Nm* and 1, this seems always to converge rapidly. Let us call this the F1 approximation.

An alternative approach that seems reasonable is, for a given pair of populations, *i* and *j*, to

- Compute *σ*^2^ = Cov[*p_i_*, *p_i_*] and do this iteratively until it converges (as before using the weighted average with weights 4*Nm* and 1). Use this for the computation of Cov[*p_i_*, *p_i_*].
- Do the same with Cov[*p_j_, p_j_*]. Use this for the computation of Cov[*p_j_*, *p_j_*].
- For the computation of Cov[*p_i_, p_j_*] use the average of these two values of *σ*^2^.

This will be referred to as the F2 approximation.

We will see that with 4*Nm* moderately large, it will make little difference which of these two methods of inferring *σ*^2^ we use. The F2 method is more tedious computationally, requiring as it does multiple iterative estimations of *σ*^2^.

## 9. Alternative equations

The covariances between populations can be arranged in a square matrix

(32) $$C=[E[(p_{i}-\bar{p})(p_{j}-\bar{p})]].$$

Using equation (2) we can show that at equilibrium **C** will satisfy

(33) $$C={\left(1-m_{\infty }\right)}^{2}MCM^{T}+Q,$$

where **Q** is the covariance matrix of *ε*, which will be diagonal. This is a linear equation in the *c_ij_*, though a big one: for example, for a 30 × 30 lattice the matrix **C** will be 900 × 900. This equation is general to any pattern of recurring migration.

It is worth noting that the linear equations in the *c_ij_* can be rewritten in a more conventional form if we convert the covariance matrices **C** and **Q** into vectors by stacking their columns into stacks s(**C**) and s(**Q**) so that

(34) $$s(C)={\left(c_{11},c_{21},c_{31},\ldots ,c_{n1},c_{12},c_{22},\ldots ,c_{2n},\ldots ,c_{1n},c_{2n},\ldots ,c_{nn}\right)}^{T}$$

and similary for s(**Q**). The equations then can be written using a Kronecker product:

(35) $$s(C)={\left(1-m_{\infty }\right)}^{2}(M⊗M)s(C)+s(Q).$$

Moving all the terms involving **C** to the left side

(36) $$\left(I-{\left(1-m_{\infty }\right)}^{2}M⊗M)s(C)=s(Q\right)$$

whereby

(37) $$s(C)={\left(I-{\left(1-m_{\infty }\right)}^{2}M⊗M\right)}^{-1}s(Q)$$

which is a slightly more general version of equation (20). The matrix inversion will always be possible if *m*_∞_ > 0.

This is a very large set of equations – for a 30 × 30 lattice of populations there will be 810,000 equations in as many unknowns. It is unlikely to be a practical numerical way of solving for the *c_ij_*, but use of a transformation analogous to equation (6),

(38) $$x=(U^{T}⊗U^{T})p,$$

allows us to solve for the covariances of **p** in a way exactly analogous to our derivation above. The approximation of the $\sigma_{i}^{2}$ is the same in this set of equations as in the previous forms.

## 10. Longer-range migration

The migration matrix allows only a single step of migration in each dimension. One straightforward way to model multiple-step migration would be to allow in each dimension a Poisson-distributed number of steps of migration, with expectation *m*. Thus, in a one-dimensional lattice a fraction

(39) $$\frac{e^{-m}m^{k}}{k!}$$

of the individuals have undergone *k* steps of migration, where each step is according to the matrix

(40) $$R=[\begin{array}{cccccc} \frac{1}{2} & \frac{1}{2} & 0 & \cdots & 0 & 0\\ \frac{1}{2} & 0 & \frac{1}{2} & \cdots & 0 & 0\\ 0 & \frac{1}{2} & 0 & \cdots & 0 & 0\\ \vdots & \vdots & \vdots & \ddots & \vdots & \vdots \\ 0 & 0 & 0 & \cdots & 0 & \frac{1}{2}\\ 0 & 0 & 0 & \cdots & \frac{1}{2} & \frac{1}{2} \end{array}].$$

As noted above in the discussion of computing the eigenvalues and eigenvectors of **M**, the matrix **R** is the special case of the one-step migration matrix with *m* = 1. The resulting migration matrix when there is a Poisson-distributed number of migration steps is

(41) $$M=\sum_{k=0}^{\infty }\frac{1}{k!}e^{-m}m^{k}R^{k}$$

This is true for any matrix **R** of one-step movements. The eigenvectors of a matrix polynomial like this are well-known to be the eigenvectors of **R**, which are the eigenvectors of the one-step migration matrix, given in equation (25). The eigenvalues of **M** in the case of a lattice can be written in terms of the eigenvalues in equation (26) – the *i*th one is

(42) $$\sum_{k=0}^{\infty }\frac{1}{k!}e^{-m}m^{k}\lambda_{i}^{k}=e^{-m}\sum_{k=0}^{\infty }\frac{1}{k!}{\left(m\lambda_{i}\right)}^{k}=e^{m(\lambda_{i}-1)}$$

If we replace *λ_i_* in this expression with the *i*th eigenvalue of the migration matrix that has *m* = 1, we get from equation (26) for the *i*th eigenvalue of the multistep migration matrix

(43) $$\lambda_{i}=\exp(m[\cos(\frac{(i-1)\pi }{n})-1])$$

For the gene frequency covariances, this can be substituted into equations (29) or (31) in place of the eigenvalue expressions there. The eigenvalues in equation (26) and those in equation (43) become asymptotically the same as *m* becomes small, as one would hope they would.

For two dimensions, tractability requires that the movement in the two dimensions be an independent random walk in each dimension. The number of steps in the *x* direction is a random Poisson variable, and so is the number of steps in the *y* direction, and these are independent. This will also be true if the total number of steps is a Poisson variate, and each step is independently chosen to be in the *x* direction or in the *y* direction, by independent Bernoulli variables (coin tosses). The eigenvalues are then all possible products of two quantities, each computed by equation (43).

## 11. Diffusion limit

It is well-known that if we consider a series of cases with increasing population sizes *N*, and decreasing values of the strengths of the deterministic evolutionary forces (here *m*_∞_ and *m*), such that the products *Nm*_∞_ and *Nm* are constant, and if we also observe the gene frequencies on a time scale in which one unit of time is *N* generations, that the stochastic process of gene frequency change approaches a diffusion process. This is of considerable interest, as the approximation is usually very close.

Taking these limits, terms in $m_{\infty }^{2}$ and *m*^2^, and all of their higher powers drop out of the equations. The result for the covariances is that equation (29) approaches the limit

(44) $$\text{Cov}[p_{i},p_{j}]=(\bar{p}(1-\bar{p})-\sigma^{2})\sum_{k=1}^{n}\frac{\Delta_{k}}{n}\cos(\frac{(k-1)(2i-1)\pi }{2n})\cos(\frac{(k-1)(2j-1)\pi }{2n})\times \frac{1}{4Nm_{\infty }+4Nm(1-\cos(\frac{(k-1)\pi }{n}))}$$

In the case of multiple-step migration, as we take *N* larger, *m* becomes small, and the occurrence of more than one step of migration becomes vanishingly rare, so it is easy to show that the diffusion limit is this same expression. The analogous expressions for the two-dimensional lattice are easily obtained and involve two terms in 4*Nm*.

## 12. Fleming and Su's approximation

Fleming and Su (1974) have derived another approximation to the finite stepping-stone models, by approximating the space as continuous. We will not give their formulas here, but below we will compare them to our approximations. I have showed elsewhere (Felsenstein, 1975) that models of finite populations of organisms migrating in continuous geographical spaces encounter some difficulties in maintaining a Poisson random field distribution. Fleming and Su's equations cannot be seen as exact for a organisms randomly distributed in a continuous geographical space. We must instead regard them as an approximation to the stepping-stone model.

To do this I have taken their interval (−ℓ, ℓ) and divided it into *n* equal intervals, with the *n* populations of the stepping-stone model being regarded as located at the midpoints of those intervals. This is to some extent an arbitrary choice.

## 13. Numerical comparisons

The approximation involved in using a common *σ*^2^ for all of the variances is expected to be a good one if 4*Nm* ≫ 1, but it is worth doing numerical checks. Table 1 shows numerical comparisons. The exact covariances for the model are calculated using equation (33), with the variances in the diagonal matrix **Q** obtained from equation (11). This equation was iterated many times until it converged.

These values, exact under our model, were compared with the approximations in equation (29). They were also compared with Maruyama's approximations in his 1970a paper, Table 4, and also with with the continuous approximation of Fleming and Su (1974). Table 1 shows the results for *n* = 10, *m*_∞_ = 0.001, *m* = 0.1, *N* = 25, and *p̄* = 0.2, the case that Maruyama considered. The exact value is denoted by E, our approximations by F1 and F2, Maruyama's approximation by M, and Fleming and Su's approximation by FS. Owing to the symmetry of the case, populations 6 through 10 have the same variances as populations 5 through 1 so they are not shown.

## 14. Numerical comparisons

The approximation involved in using a common *σ*^2^ for all of the variances is expected to be a good one if 4*Nm* ≫ 1, but it is worth doing numerical checks. Table 1 shows numerical comparisons. The exact covariances for the model are calculated using equation (33), with the variances in the diagonal matrix **Q** obtained from equation (11). This equation was iterated many times until it converged.

These values, exact under our model, were compared with the approximations in equation (29). They were also compared with Maruyama's approximations in his 1970a paper, Table 4, and also with with the continuous approximation of Fleming and Su (1974). Table 1 shows the results for *n* = 10, *m*_∞_ = 0.001, *m* = 0.1, *N* = 25, and *p̄* = 0.2, the case that Maruyama considered. For Fleming and Su's approximation, which uses a continuous space on the interval (−1, 1), the locations corresponding to the *n* populations were taken to be $1-\frac{1}{n},1-\frac{3}{n},1-\frac{5}{n},\ldots ,-\frac{1}{n},\frac{1}{n},\ldots ,1-\frac{1}{n}$. These are *n* points equally spaced, each being the center of an interval which is $\frac{1}{n}$ of the total interval, which is of length 2. The exact value is denoted by E, our approximations by F1 and F2, Maruyama's approximation by M, and Fleming and Su's approximation by FS. Owing to the symmetry of the case, populations 6 through 10 have the same variances as populations 5 through 1 so they are not shown.

While generally similar, then two approximations differ noticeably, especially for the terminal population. In Maruyama's approximation, there is an extra injection of mutation into the terminal populations. This damps its departure from the mutational equilibrium which is caused by genetic drift. Both approximations are considerably lower than the true variance, the expected variance in the F1 approximation developed in this paper being 18% low in the terminal population and 26% low in the central population. The F2 approximation is slightly worse in the populations near the ends, and slightly better in the central region, but perhaps not enough to make it worthwhile in view of its higher computational burden. The Fleming-Su approximation is worse. It does agree with the other values by showing higher variance in the terminal populations.

Table 1 has 4*Nm* = 10 and 4*Nm*_∞_ = 0.1. When 4*Nm* = 100, as in Table 2, the F1 approximation is much better, being 0.56% high in the terminal populations, and 0.3% low in the center populations. The F2 approximation is again lower than the F1 approximation in the end region and higher than it in the central region; in most populations it is farther from the exact value than is the F1 approximation.

In longer one-dimensional stepping stone models, as in Table 3, we can see that the exact variances in the terminal populations are higher than in the center, but that this rapidly declines and becomes relatively constant as we move toward the center of the lattice. The F1 approximation tracks this quite closely, being about 3.6% too high in the terminal populations and 0.5% too low in the center. The F2 approximation is in general closer to the exact value, and through most of the central region is nearly exact. The Fleming-Su approximation does even less well than before.

We can also compare the correlations between the gene frequencies of pairs of populations. Table 4 shows these for the same case as Table 1 above, where *N* = 25. For this case Maruyama also calculated the correlations from his reflecting boundary approximation, in Table 4 of his 1970b paper. Both of our approximations are very close, and his quite a bit farther away. The (1,9) element of this table has the greatest departure from the exact value for both our and Maruyama's approximation, and his is 8.24% high while ours is 0.67% low. (There is a typographical error in the Theoretical number in the (9,10) element of his table). The F2 approximation is slightly better than the F1 approximation for pairs of populations near the ends of the region, and slightly better for all other pairs.

Table 5 shows the correlations for the case when *N* = 250. It can be shown that the F1 approximation will not depend on *N*, so our predictions are the same as in Table 4. In fact, the expressions for predicted correlations using our approximation also do not depend on *p̄* or on *m*_∞_ either – they depend only on the geometry of the populations and on *m*. Compared to these predictions, the exact values are a bit more different in Table 5, being up to 5% low (in the case of the (10,1) element), but more typically being about 1% low. The F2 approximations do depend on *N*, but for the correlations they are so close to the values of the F1 approximation that there seems little point going to the extra effort of computing them.

## 15. Usefulness

The present solution is approximate, but less so that previous efforts. It is intended for use in a likelihood-based (or Bayesian-based) inference of migration and/or mutation parameters, scaled as a fraction of the local effective population size. However the approximation is specific to rectangular lattices which have reached equilibrium between mutation, migration, and genetic drift. Both aspects, the rectangularity and the equilibrium, may be questioned for natural populations, the equilibrium being a particularly severe challenge for human populations. In continents such as Europe, we need assurance that there has been enough time since historical population movements such as those associated with the spread of agriculture. Given perhaps only 7000 years since the establishment of agriculture there, that is a stringent requirement.

The availability of approximations based on processes in a continuum of finite length, instead of the stepping stone lattice, presents another challenge. We have compared our formulas for the variances with Fleming and Su's (1974) approximation, and for these cases the continuous approximation was a worse approximation. Barton and Wilson (1995) have developed approximations for coalescent times for an approximate model of a continuum of finite length. The papers by Barton et al. (2002, 2010) make similar approximations that allow for extinction and recolonization, which have not been discussed here. These papers use, respectively, a model of a two-dimensional infinite continuum, and a model of a finite torus. Wilkins and Wakeley (2002) and Wilkins (2004) use a different approximate model to develop other approximations to coalescence times in a one- or two-dimensional finite continuum. Neither of these approximations has been developed into an approximation for variances and covariances of local gene frequencies, although it does not seem difficult to do so. Barton and Wilson (1995) approximate these covariances by using Malécot's (1951) formulae for the one- and two-dimensional infinite continuum approximation.

If the stepping-stone model is regarded as the truth, and these continuum models are intended as approximations to it, the present formulas may help evaluate the accuracy of the continuum approximations. But to the extent that both the stepping-stone models and the continuum models are regarded as approximations to the more subtle structure of real populations, we would have to model those, perhaps in individual-based simulations, to evaluate whether either approach is viable.

## Figures and Tables

**Table 1:** Comparison of exact solution (E) for the variances of population gene frequencies in a 10-stone stepping-stone model for *n* = 10, *m*_∞_ = 0.001, *m* = 0.1, *N* = 25, and *p̄* = 0.2 with the approximations of this paper (F1 and F2), Maruyama's reflecting-boundary approximation (M), and Fleming and Su's (1974) approximation. Approximation F1 uses the value of *σ*^2^ averaged over all populations. Approximation F2 uses a value of *σ*^2^ calculated for that population. As the migration pattern is symmetrical, only the values for populations 1-5 are shown.

population	E	F1	F2	M	FS
1	0.1281	0.1046	0.0961	0.0982	0.0811
2	0.1161	0.0946	0.0922	0.0928	0.0720
3	0.1121	0.0877	0.0892	0.0879	0.0665
4	0.1107	0.0834	0.0872	0.0857	0.0634
5	0.1103	0.0814	0.0862	0.0846	0.0619

**Table 2:** Comparison of exact solution (E) for the variances of population gene frequencies in a 10-stone stepping-stone model for *n* = 10, *m*_∞_ = 0.001, *m* = 0.1, *N* = 250, and *p̄* = 0.2 with the approximation of this paper (F). Approximation F1 uses the value of *σ*^2^ averaged over all populations. Approximation F2 uses a value of *σ*^2^ calculated for that population. FS is the Fleming-Su approximation. As the migration pattern is symmetric, only the values for populations 1-5 are shown.

population	E	F1	F2	FS
1	0.02115	0.02127	0.02089	0.01396
2	0.01918	0.01924	0.01913	0.01207
3	0.01784	0.01784	0.01790	0.01087
4	0.01700	0.01697	0.01712	0.01016
5	0.01660	0.01655	0.01674	0.00984

**Table 3:** Comparison of exact solution (E) for the variances of population gene frequencies in a 100-stone stepping-stone model for *n* = 100, *m*_∞_ = 0.001, *m* = 0.1, *N* = 250, and *p̄* = 0.2 with the approximations of this paper (F1 and F2). Approximation F1 uses the value of *σ*^2^ averaged over all populations. Approximation F2 uses a value of *σ*^2^ calculated for that population. As the migration pattern is symmetric, only values for the first half of the lattice are shown.

population	E	F1	F2
1	0.01900	0.01968	0.01870
2	0.01691	0.01744	0.01679
3	0.01534	0.01575	0.01532
4	0.01416	0.01447	0.01419
5	0.01328	0.01351	0.01333
10	0.01126	0.01139	0.01129
20	0.01066	0.01061	0.01066
30	0.01062	0.01057	0.01062
40	0.01062	0.01057	0.01062
50	0.01062	0.01057	0.01062

**Table 4:** Exact solution and two approximations for the correlations of population gene frequencies for *n* = 10, *m*_∞_ = 0.001, *m* = 0.1, *N* = 25, and *p̄* = 0.2. Within each nondiagonal cell, the uppermost value is the exact solution, the next two values our F1 and F2 approximations, and the lower value is Maruyama's reflecting-boundary approximation, from Table 4 of his 1970b paper.

	1	2	3	4	5	6	7	8	9	10
1	1.0									
2	0.9292	1.0								
	0.9286									
	0.9290									
	0.9410									
3	0.8607	0.9223	1.0			Exact				
	0.8600	0.9223				F1				
	0.8611	0.9225				F2				
	0.8795	0.9264				M				
4	0.7931	0.8499	0.9171	1.0						
	0.7924	0.8499	0.9174							
	0.7940	0.8504	0.9175							
	0.8175	0.8586	0.9209							
5	0.7287	0.7808	0.8424	0.9139	1.0					
	0.7279	0.7807	0.8428	0.9144						
	0.7297	0.7814	0.8430	0.9144						
	0.7541	0.7928	0.8496	0.9177						
6	0.6690	0.7167	0.7731	0.8386	0.9128	1.0				
	0.6679	0.7164	0.7734	0.8392	0.9134					
	0.6696	0.7170	0.7736	0.8392	0.9134					
	0.6971	0.7315	0.7834	0.8455	0.9166					
7	0.6152	0.6589	0.7106	0.7705	0.8386	0.9139	1.0			
	0.6136	0.6581	0.7105	0.7709	0.8392	0.9144				
	0.6149	0.6585	0.7106	0.7709	0.8392	0.9144				
	0.6427	0.6756	0.7233	0.7804	0.8455	0.9177				
8	0.5676	0.6080	0.6555	0.7106	0.7731	0.8424	0.9171	1.0		
	0.5655	0.6065	0.6548	0.7105	0.7734	0.8428	0.9174			
	0.5662	0.6066	0.6548	0.7106	0.7736	0.8430	0.9175			
	0.5974	0.6268	0.6709	0.7233	0.7834	0.8496	0.9209			
9	0.5265	0.5639	0.6080	0.6589	0.7167	0.7808	0.8499	0.9223	1.0	
	0.5238	0.5618	0.6065	0.6581	0.7164	0.7807	0.8499	0.9223		
	0.5240	0.5618	0.6066	0.6585	0.7170	0.7814	0.8504	0.9225		
	0.5571	0.5855	0.6268	0.6756	0.7315	0.7928	0.8586	0.9264		
10	0.4916	0.5265	0.5676	0.6152	0.6690	0.7287	0.7931	0.8607	0.9292	1.0
	0.4883	0.5238	0.5655	0.6136	0.6679	0.7279	0.7924	0.8600	0.9286	
	0.4883	0.5240	0.5662	0.6149	0.6696	0.7297	0.7940	0.8611	0.9290	
	0.5321	0.5571	0.5974	0.6427	0.6971	0.7541	0.8175	0.8795	0.9410	

**Table 5:** Comparison of exact solution (E) for the correlations of population gene frequencies in a 10-stone stepping-stone model for *n* = 10, *m*_∞_ = 0.001, *m* = 0.1, *N* = 250, and *p̄* = 0.2 with the approximation of this paper. The 10 × 10 lower triangle contains three numbers, the uppermost one being the exact correlation and the lower two our approximations F1 and F2. Approximation F1 is calculated with one value of *σ*^2^ for all populations; approximation F2 is calculated using variances from approximations of *σ*^2^ local to the two populations involved, and an average of these values is used to compute the covariance between the two populations.

	1	2	3	4	5	6	7	8	9	10
1	1.0									
2	0.9332	1.0								
	0.9286									
	0.9287									
3	0.8657	0.9226	1.0			Exact				
	0.8600	0.9223				F1				
	0.8600	0.9223				F2				
4	0.7985	0.8501	0.9151	1.0						
	0.7924	0.8499	0.9174							
	0.7925	0.8499	0.9174							
5	0.7350	0.7816	0.8398	0.9106	1.0					
	0.7279	0.7807	0.8428	0.9144						
	0.7279	0.7807	0.8428	0.9144						
6	0.6770	0.7193	0.7715	0.8346	0.9092	1.0				
	0.6679	0.7164	0.7734	0.8392	0.9134					
	0.6680	0.7164	0.7734	0.8392	0.9134					
7	0.6259	0.6645	0.7115	0.7681	0.8346	0.9106	1.0			
	0.6136	0.6581	0.7104	0.7709	0.8392	0.9144				
	0.6136	0.6581	0.7105	0.7709	0.8392	0.9144				
8	0.5820	0.6174	0.6603	0.7115	0.7715	0.8398	0.9151	1.0		
	0.5655	0.6065	0.6548	0.7104	0.7734	0.8428	0.9174			
	0.5655	0.6065	0.6548	0.7105	0.7734	0.8428	0.9174			
9	0.5449	0.5778	0.6174	0.6645	0.7193	0.7816	0.8501	0.9226	1.0	
	0.5238	0.5618	0.6065	0.6581	0.7164	0.7807	0.8499	0.9223		
	0.5238	0.5618	0.6065	0.6581	0.7164	0.7807	0.8499	0.9223		
10	0.5140	0.5449	0.5820	0.6259	0.6770	0.7350	0.7985	0.8657	0.9332	1.0
	0.4883	0.5238	0.5655	0.6136	0.6679	0.7279	0.7924	0.8600	0.9286	
	0.4883	0.5238	0.5655	0.6136	0.6680	0.7279	0.7925	0.8600	0.9287	

## Appendix

Feller (1957, section XVI.3, p. 390) gives the elements of the *i*th right eigenvector of matrix **M** when *m* = 1 as

(45) $$u_{ij}=\sin(\frac{(i-1)j\pi }{n})-\sin(\frac{(i-1)(j-1)\pi }{n}),$$

where they are determined up to an arbitrary multiplicative constant.

Note the trigonometric relationship

(46) sin(θ+ϕ)+sin(θ−ϕ)=2cos(θ)sin(ϕ)

which may readily be verified using the addition law for sines.

Taking

(47) $$\theta =\frac{1}{2}(\frac{(i-1)j\pi }{n}+\frac{\left(i-1)(j-1\right)}{n})=\frac{(i-1)(2j-1)\pi }{2n}$$

and

(48) $$\phi =\frac{1}{2}(\frac{(i-1)j\pi }{n}-\frac{(i-1)(j-1)\pi }{n})=\frac{(i-1)\pi }{2n}$$

we find that the elements of the eigenvector can be written as

(49) $$u_{ij}=\cos(\frac{(i-1)(2j-1)\pi }{2n})\sin(\frac{(i-1)\pi }{2n})$$

The sine factor does not depend on *j*, so it is a scaling of the *i*th eigenvector. These eigenvectors each need to be rescaled so that the eigenvectors are orthonormal. If they are written as

(50) $$u_{ij}=\sqrt{\frac{\Delta_{i}}{n}}\cos(\frac{(i-1)(2j-1)\pi }{2n})$$

where Δ*_i_*, is 1 for *i* = 1 and 2 otherwise, it can be shown that this is the appropriate scaling.
